# Chronic prenatal delta-9-tetrahydrocannabinol exposure adversely impacts placental function and development in a rhesus macaque model

**DOI:** 10.1038/s41598-022-24401-4

**Published:** 2022-11-24

**Authors:** Victoria H. J. Roberts, Matthias C. Schabel, Emily R. Boniface, Rahul J. D’Mello, Terry K. Morgan, Juanito Jose D. Terrobias, Jason A. Graham, Laura M. Borgelt, Kathleen A. Grant, Elinor L. Sullivan, Jamie O. Lo

**Affiliations:** 1 Division of Reproductive and Developmental Sciences, Oregon National Primate Research Center, Oregon Health & Science University, Beaverton, OR USA; 2 Advanced Imaging Research Center, Oregon Health & Science University, Portland, OR USA; 3 Department of Obstetrics and Gynecology, Oregon Health & Science University, 3181 SW Sam Jackson Park Road, Mail Code L458, Portland, OR 97239 USA; 4 Department of Pathology, Oregon Health & Science University, Portland, OR USA; 5 Division of Neuroscience, Oregon National Primate Research Center, Oregon Health & Science University, Beaverton, OR USA; 6 Department of Clinical Pharmacy and Family Medicine, University of Colorado Anschutz Medical Campus, Aurora, CO USA; 7 Department of Psychiatry, Oregon Health & Science University, Portland, OR USA

**Keywords:** Experimental models of disease, Preclinical research, Translational research

## Abstract

Cannabis use in pregnancy is associated with adverse perinatal outcomes, which are likely mediated by the placenta. However, the underlying mechanisms and specific vasoactive effects of cannabis on the placenta are unknown. Our objective was to determine the impact of chronic prenatal delta-tetrahydrocannabinol (THC, main psychoactive component of cannabis) exposure on placental function and development in a rhesus macaque model using advanced imaging. Animals were divided into two groups, control (CON, n = 5) and THC-exposed (THC, n = 5). THC-exposed animals received a THC edible daily pre-conception and throughout pregnancy. Animals underwent serial ultrasound and MRI at gestational days 85 (G85), G110, G135 and G155 (full term is ~ G168). Animals underwent cesarean delivery and placental collection at G155 for histologic and RNA-Seq analysis. THC-exposed pregnancies had significantly decreased amniotic fluid volume (p < 0.001), placental perfusion (p < 0.05), and fetal oxygen availability (p < 0.05), all indicators of placental insufficiency. Placental histological analysis demonstrated evidence of ischemic injury with microinfarctions present in THC-exposed animals only. Bulk RNA-seq demonstrated that THC alters the placental transcriptome and pathway analysis suggests dysregulated vasculature development and angiogenesis pathways. The longer-term consequences of these adverse placental findings are unknown, but they suggest that use of THC during pregnancy may deleteriously impact offspring development.

## Introduction

Due to recent and widespread legalization at the state level, prenatal cannabis use has more than doubled in the past decade^1^ and it is now the most common illicit drug used by pregnant individuals^2,3^. This high prevalence of prenatal cannabis use is in part because approximately half of all pregnancies are unplanned and most women do not recognize they are pregnant until 4 to 6 weeks post conception^4^. First trimester use of cannabis to treat early pregnancy nausea is common, and coincides with a developmental window (including organogenesis) when the fetus is most vulnerable to adversity. The American College of Obstetricians and Gynecologists^5^, and the American Academy of Pediatrics^6^ advise pregnant and lactating patients to abstain from cannabis. Despite these recommendations, many patients continue to use^7–9^, in part because safety data is lacking and the available literature is insufficient to establish a casual association between cannabis use and negative antenatal outcomes^5,10^.

The main psychoactive component of cannabis, delta-9-tetrahydrocannabinol (THC), readily crosses the placenta and binds to endocannabinoid receptors in the placenta and key fetal organ systems, leading to concern for detrimental effects on placental and fetal development^11–15^. Existing studies suggest an increased risk of adverse effects with prenatal cannabis exposure that include preterm birth, stillbirth, small for gestational age infants, and altered offspring neurodevelopment^2,13,15–21^, but are limited by weak mechanistic understanding and reliance on patient self-reporting. Results are inconsistent among studies, with most studies failing to control for important confounders such as tobacco use. Consequently, the underlying etiology of these adverse outcomes is unclear.

The placenta occupies a critical role in normal maternal–fetal oxygen and nutrient exchange, and altered placental function can result in abnormal fetal development. Rodent placentas exposed to daily intraperitoneal injections of THC exhibited a phenotype characterized by vascular defects including decreased fetal capillary area^22^. Human data demonstrate that maternal cannabis use is associated with decreased expression of genes involved in placental immune system function, and that several genes organize into co-expression networks that correlate with child anxiety and hyperactivity^23^.

To address key gaps in the evidence and overcome the limitations of previous human studies, we built upon our novel non-human primate (NHP) model of chronic cannabis exposure via edible THC consumption^24,25^. Edibles are the second most common mode of cannabis delivery^26^, especially in non-daily pregnant users^27^, and are often recommended by dispensaries to pregnant individuals for nausea^28^. NHPs and humans have similar fetal ontogeny^29,30^, placental structure^29,30^, and THC plasma disposition, resulting in observations that are directly translatable to human pregnancies^31^. A NHP model allows a longitudinal study design, standardization of subject variability, and precise THC dosing to elucidate direct biological consequences of chronic prenatal cannabis exposure while methodically controlling for potential confounders.

In combination, we leveraged our previously developed novel non-invasive MRI methods for the assessment of in utero placental function. We have successfully detected disruption of placental function and development in response to other environmental perturbations, including maternal substance use, in vivo^32–36^. Placental blood flow is measured using dynamic contrast-enhanced MRI (DCE-MRI)^37^ and oxygen exchange is quantified through analysis of water *T*_2_* values via the blood oxygen level-dependent (BOLD) effect^35^.

Prenatal cannabis use and potency are increasing, therefore, the need for evidence-driven recommendations on the safety of use during pregnancy is urgent. The primary objective of our study was to evaluate the adverse effects of chronic, prenatal cannabis exposure on placental function and development using a first-in-kind rhesus macaque model of maternal, contemporary THC edible use. Our second objective was to study the effects of prenatal cannabis exposure on placental histology and gene expression to identify mechanisms underlying placental dysfunction.

## Materials and methods

### Experimental design

All protocols were approved by the Oregon National Primate Research Center (ONPRC) Institutional Animal Care and Use Committee and conformed to all guidelines for humane animal care (IP0001389). Methods are reported in accordance with the ARRIVE guidelines (https://arriveguidelines.org)^38^. This study used indoor-housed rhesus macaques (n = 10) maintained on a standard chow diet (TestDiet, St. Louis, Missouri). Cookies containing THC (THC edible) were made using research-grade THC obtained directly from the National Institute of Drug Abuse (NIDA)^24,25^.

**Figure 1:**
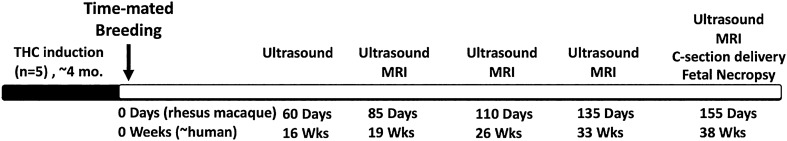
Study design overview. Timeline of the experimental design indicating that the THC-exposed animals underwent ~ 4 months of THC induction preconception with incremental dosing increase until reaching a dose of 2.5 g/7 kg/day (approximately a heavy medical cannabis dose) that was then maintained throughout pregnancy. The age-matched control group were administered a daily edible without THC in addition to their daily chow. Ultrasound was performed at (G60, term is ~ 168 days), and both US and MRI were performed at G85, G110 and G135, and G155 with immediate cesarean delivery following imaging at G155.

Tap water was available ad libitum. Edibles were administered prior to the animal's daily chow to ensure consumption on an empty stomach and to confirm complete ingestion. Animals were slowly titrated up to 2.5 mg/7 kg/day of THC using published weight-based medical cannabis acclimation recommendations^39^ approximately 4 months prior to undergoing time-mated breeding as previously published^24^. Each THC-exposed pregnant animal (n = 5) continued to consume a daily edible of 2.5 mg/7 kg/day throughout pregnancy. All animals (n = 10) underwent Doppler-(D-US) and Contrast-enhanced ultrasound (CE-US) on gestational days 60, 85, 110, 135 and 155 (Fig. 1). Placental MRI consisting of *T*_2_* and DCE measurements was performed following ultrasound studies on gestational days 85, 110, 135 and 155. On gestational day 155, after imaging, animals underwent immediate cesarean section delivery with placenta collection and fetal necropsy (Fig. 1). Collected placental tissue was processed in RNAlater for RNA-sequencing, and formalin fixation for histology.

### Imaging

#### Doppler-ultrasound

Ultrasounds were performed by a single sonographer (J.O.L) using image-directed pulsed and color Doppler equipment (GE Voluson 730) with a 5- to 9-MHz sector probe. Animals were sedated by intramuscular administration of 10 mg/kg ketamine (Henry Schein Animal Health^®^) and maintained on 1.5% isoflurane. Amniotic fluid volume was measured per standard clinical protocol by dividing the uterine cavity into four quadrants, the largest vertical diameter in each quadrant was measured and the sum of the quadrants provided the amniotic fluid index. Doppler waveform measurements for the uterine artery (Uta) and umbilical artery were performed using machine-specific software. The following measurements were obtained: pulsatility index (PI), velocity time integral (VTI), and fetal heart rate (HR) to calculate uterine artery blood flow (cQ_Uta_) and placental volume blood flow (cQ_UV_) as previously described^33,34,40–43^. cQ_Uta_ was calculated and corrected by maternal weight as: cQ_Uta_ = VTI × CSA (Uta cross-sectional area) × HR^40–43^. Placental volume blood flow (cQ_UV_) was calculated as: mean velocity (V_mean_) × CSA × 60^40–43^.

### Contrast-enhanced ultrasound

Contrast-enhanced ultrasound (CE-US) was performed using a multiphase amplitude modulation and phase-inversion algorithm on a Sequoia system (Siemens Medical Systems, Mountain View, California) equipped with a 15L8 transducer at a transmit frequency of 7 MHz with a 0.18 mechanical index (MI) and a 55 dB dynamic range with intravenous administration of lipid-shelled octafluoropropane microbubble contrast reagent (Definity; Lantheus Medical Imaging, Billerica, Massachusetts)^44^. Three replicates of all recordings were obtained and digital imaging data were analyzed as previously described in detail^44,45^. In brief, regions of interest were drawn over the intervillous space perfused by 1 maternal spiral artery input source. Replenishment kinetic curves were generated in a custom software program, and microvascular flux rate (β) was calculated as the rate of refilling of the vascular space until signal saturation was reached.

### Placental MRI

Immediately following ultrasound, MRI was performed on a 3 T Siemens TIM-Trio scanner (Erlangen, Germany) with continuous physiological monitoring as previously published^33,34^. Following localization of the placenta and acquisition of anatomic, axial 2D multislice multiecho spoiled gradient echo images spanning the entire uterus were acquired at six in-phase echo times for T2* quantification, and ${T}_{1}$ was measured with the variable flip angle (VFA) method^46^. After acquisition of VFA data, 150 3D SPGR volumes were acquired for DCE-MRI with intravenous injection of 0.1 mmol/kg of gadoteridol contrast reagent (Prohance^®^, Bracco Diagnostics Inc, Princeton, NJ), and with field of view and resolution matched to the VFA images. BOLD and DCE-MRI analyses were performed as previously described^32–34,46^.

### Placental histology

Formalin fixed paraffin-embedded histologic sections were stained with hematoxylin and eosin and reviewed by a single placental pathologist (T.K.M.) blinded to exposure and outcomes. Tissue sections were scored for any signs of infection and classic histologic features of maternal vascular malperfusion, including infarctions and/or accelerated villous maturation^47^.

### Statistical analysis

Mean (± standard deviation) of all fetal biometry and ultrasound measurements were reported at each timepoint and the average changes over time were estimated using linear mixed effects modeling with random intercepts by animal. Treatment group differences in mean maternal age, maternal weight at G60 and G155, fetal birthweight, fetal tissue weight, and total placental weight were assessed using Welch’s t-test. All statistical tests were two-sided and used an alpha of 0.05. Analyses were performed using Stata^®^ version 15.1 (StataCorp, College Station, TX). Linear regression analysis was performed for CE-US data replicates. Differences in DCE-MRI results and BOLD-MRI *T*_2_* results were evaluated for a THC effect by repeated measures ANOVA.

### Gene expression

#### RNA isolation and quality assessment

Dissected placental tissue samples (n = 10) in RNAlater (ThermoFisher Scientific) were processed by the OHSU Gene Profiling Shared Resource, where phenol–chloroform extraction was performed followed by RNA isolation using the RNeasy Mini kit (QIAGEN). RNA integrity and size distribution were assessed using a 2100 Bioanalyzer (Agilent Technologies) as previously published^34^.

### RNA sequencing and gene-level differential expression analysis

Isolated placental RNA were sequenced by Novogene. A Poly-A enrichment step was used to select for mRNA molecules followed by fragmentation, reverse transcription, and Illumina-compatible adaptor ligation (PCR and library construction). Libraries were sequenced on a NovaSeq 6000 S4 flow cell (PE150) to generate 20 million paired reads. For bioinformatic analysis, the raw data was filtered and mapped to the rhesus macaque genome. Gene expression levels were quantified using FPKM values to screen for differentially expressed genes (DEGs). ClusterProfiler software^48^, including Gene Ontology enrichment analysis and KEGG pathway enrichment analysis was performed^49–51^, on DEGs. Pathway analysis was performed using GO terms and KEGG terms with an adjusted p-value < 0.05.

### Ethical approval and consent to participate

The study was approved by the Ethics Committee of the Oregon National Primate Center.

### Conference presentation

Presented as oral presentations at the 69th Society of Reproductive Investigation Annual Meeting March 17–19th, 2022 in Denver, Colorado and as poster presentations at the virtual 68th Society of Maternal Fetal Medicine Annual Meeting, February 3–5th, 2022.

## Results

### THC measures and growth parameters

During the THC induction, average plasma THC concentrations increased by 4.5 ng/mol for each mg/7 kg/day increase in THC (95% CI: 2.6–6.5 ng/mol, p < 0.001). Peak THC levels at the highest THC dosing regimen were within the expected reported contemporary dosing range (e.g., 5–8 ng/mL) in humans 3 h following a similar oral THC dose^52,53^. Ultrasound measurements of fetal biometry were similar between fetuses exposed to THC and controls across pregnancy (Table 1), and average changes per 25 days of gestation were not significantly different between treatment groups. There were no statistical significance differences in fetal birth weight, maternal weights or fetal gender ratios across treatment groups (Table 2). Fetal tissue weights and tissue:fetal weight ratios were similar between treatment groups, except in THC-exposed fetuses where testicular weight was decreased by approximately half (p < 0.05), and a decreased heart to fetal weight ratio was reported (p < 0.05) (Table 2).

**Table 1:** Doppler and contrast-enhanced ultrasound measurements by gestational age timepoint.

Variable	Group	60 days	85 days	110 days	135 days	155 days
Biparietal diameter (cm)	Control	1.93 ± 0.14	2.87 ± 0.22	3.86 ± 0.24	4.52 ± 0.26	4.80 ± 0.07
	THC	1.87 ± 0.13	3.03 ± 0.19	3.90 ± 0.22	4.37 ± 0.30	4.72 ± 0.17
Head circumference (cm)	Control	7.15 ± 0.27	10.67 ± 0.85	14.46 ± 0.87	16.88 ± 0.76	18.00 ± 0.46
	THC	6.81 ± 0.36	11.01 ± 0.54	14.70 ± 0.64	16.69 ± 0.75	17.56 ± 0.64
Abdominal circumference (cm)	Control	5.61 ± 0.42	8.87 ± 0.80	11.85 ± 0.39	13.54 ± 1.16	15.06 ± 0.51
	THC	5.56 ± 0.66	8.94 ± 0.51	11.65 ± 0.62	12.91 ± 0.71	14.13 ± 1.04
Femur length (cm)	Control	0.80 ± 0.11	1.81 ± 0.27	2.85 ± 0.26	3.44 ± 0.27	3.84 ± 0.11
	THC	0.68 ± 0.13	1.81 ± 0.25	2.72 ± 0.19	3.40 ± 0.07	3.72 ± 0.29
Placental volume blood flow (cQ_UV_/kg)	Control	0.22 ± 0.12	0.80 ± 0.35	1.47 ± 0.64	2.19 ± 0.47	2.17 ± 0.70
	THC	0.11 ± 0.04	0.79 ± 0.22	1.38 ± 0.42	1.98 ± 0.42	2.14 ± 0.90
Uterine artery blood flow (cQ_uta_/kg)	Control	0.14 ± 0.04	0.10 ± 0.01	0.12 ± 0.04	0.12 ± 0.04	0.12 ± 0.06
	THC	0.07 ± 0.05	0.16 ± 0.12	0.18 ± 0.07	0.17 ± 0.11	0.11 ± 0.10
Contrast enhanced ultrasound (ms^−1^)	Control	0.19 ± 0.04	0.22 ± 0.04	0.21 ± 0.05	0.25 ± 0.11	0.22 ± 0.08
	THC	0.23 ± 0.03	0.23 ± 0.03	0.21 ± 0.02	0.19 ± 0.06	0.21 ± 0.03
Umbilical artery pulsatility index (PI)	Control	2.70 ± 0.46	2.09 ± 0.28	1.69 ± 0.16	1.39 ± 0.31	1.09 ± 0.17
	THC	2.41 ± 0.43	1.78 ± 0.24	1.64 ± 0.41	1.32 ± 0.34	1.30 ± 0.50

CSA (cross section of uterine artery) = π(diameter/2)^2^.

Vmean (mean velocity) = 0.5 × maximum umbilical vein velocity.

cQuta (uterine artery blood flow) = VTI × CSA × HR adjusted for maternal weight.

cQuv (placental volume blood flow) = Vmean × CSA × 60.

*BPD* biparietal diameter, *AC* abdominal circumference, *FL* femur length, *PI* pulsatility index, *VTI*  velocity time integral.

**Table 2:** Maternal, fetal, and placental weights and demographics.

Characteristic	Control	THC	p-value
N	5	5	
Maternal age (years)	10.0 ± 2.3	9.9 ± 2.1	0.945
Parity	3.6 ± 0.5	2.2 ± 2.2	0.226
Maternal weight at G60 days (kg)	7.53 ± 1.02	7.74 ± 0.47	0.692
Maternal weight at G155 days (kg)	8.94 ± 1.20	8.95 ± 0.48	0.990
Total placental weight (g)	105.2 ± 17.6	105.0 ± 18.7	0.984
**Fetal measurements**			
Fetal sex (male:female)	2:3	3:2	–
Fetal birth weight (kg)	0.48 ± 0.08	0.46 ± 0.07	0.676
CRL (cm)	19.12 ± 1.20	19.32 ± 2.08	0.859
Right foot (cm)	7.62 ± 0.66	7.3 ± 0.56	0.434
AC (cm)	19.32 ± 2.08	11.92 ± 1.57	0.930
HC (cm)	17.18 ± 4.84	19.04 ± 0.98	0.444
Brain (g)	53.28 ± 2.71	51.92 ± 1.69	0.373
Thymus (g)	1.92 ± 0.59	1.45 ± 0.37	0.177
Thyroid (g)	0.35 ± 0.10	0.20 ± 0.09	0.067
Adrenals (g)	0.26 ± 0.04	0.29 ± 0.13	0.598
Pituitary (g)	0.01 ± 0.01	0.02 ± 0.01	0.683
Ovaries (g)	0.09 ± 0.01 (n = 3)	0.09 ± 0.08 (n = 2)	0.981
Testes (g)*	0.13 ± 0.10 (n = 2)	0.06 ± 0.03 (n = 3)	< 0.05
Kidneys (g)	2.14 ± 0.31	2.18 ± 0.23	0.875
Pancreas (g)	0.35 ± 0.07	0.39 ± 0.18	0.643
Liver (g)	12.00 ± 2.23	12.32 ± 2.39	0.831
Lungs (g)	9.45 ± 2.76	9.07 ± 3.16	0.845
Spleen (g)	0.73 ± 0.17	0.57 ± 0.23	0.249
Heart (g)	2.84 ± 0.47	2.38 ± 0.62	0.222
Body weight (g)	484.36 ± 76.51	463.71 ± 71.21	0.670
Heart to weight ratio*	0.0058 ± 0.0005	0.0051 ± 0.0006	< 0.05

*CRL* crown rump length, *AC* abdominal circumference, *HC* head circumference.

Tissue to fetal body weight ratio was not significant for all tissues except heart.

Data are means ± SD. Statistical analysis performed using Welch’s t-test.

*p < 0.05.

### Amniotic fluid volume

**Figure 2:**
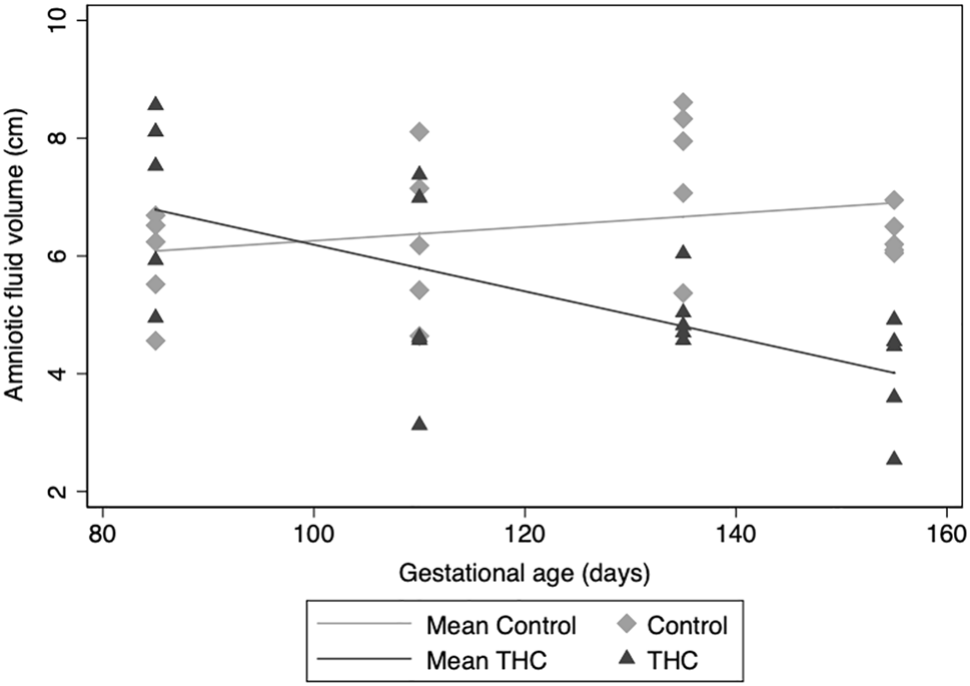
Amniotic fluid volume across gestation. Individual amniotic fluid volume measurements at four gestational time points. With increasing gestation, we observed a significant decrease in average change per 25 days of gestation in amniotic fluid volume in the THC-exposed (n = 5) vs. control (n = 5) pregnancies (p < 0.001).

An expected increase in amniotic fluid volume across pregnancy was observed in control animals (Fig. 2), but a statistically significant decrease in amniotic fluid volume throughout pregnancy was present in THC-exposed animals (p < 0.001).

### Placental perfusion and oxygenation

The umbilical artery PI decreased with gestational age as expected and did not deviate from control in any of the THC-exposed animals (Table 1). Quantitative estimation of placental volumetric blood flow in the uterine artery has been previously used by our group and others^33,34,40–43,54^ to assess maternal-side placental perfusion. cQuta was not significantly different in the THC-exposed group compared to controls across gestation (Table 1). Similarly, the quantitative estimation of blood flow on the fetal side of the placenta^33,34,43,54^, the cQuv, increased across gestation as expected in both THC-exposed animals and control animals with no significant differences between groups (Table 1). We employed CE-US to visualize and quantify perfusion in the placental intervillous space (IVS). The flux rate constant (*β*) provides a measure of microvascular resistance and gives an indirect measure of blood flow in the IVS^55^. In comparing *β*, there was no difference in vascular impedance between THC-exposed and control animals (Table 1).

**Figure 3:**
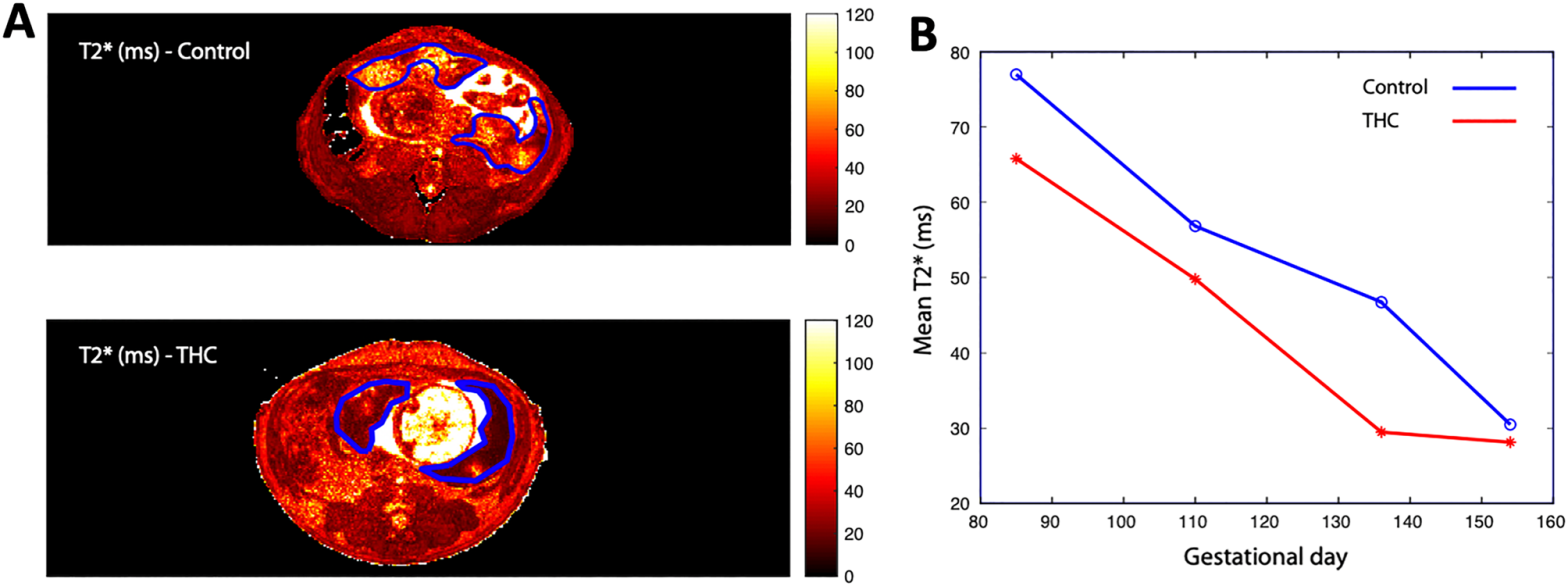
Histogram plot of *T*_2_* versus percent of placental voxels displayed for THC-exposed (red) vs. control animals (blue) at G85, G110, G135 and G155. (**A**) Quantitative *T*_2_* maps of placental blood flow of an axial MRI-image at the level of the uterus of a representative control (top) and THC-exposed (bottom) animal with the bi-lobed rhesus macaque placenta outlined in blue. (**B**) THC-exposed animals had a smaller fraction of large *T*_2_* values compared to controls across all time points, demonstrating decreased placental perfusion and fetal oxygen availability in the former. *p = 0.04 for THC effect by repeated measures ANOVA.

For more comprehensive quantification of blood flow across the entire placenta, DCE-MRI was utilized to assess maternal perfusion of the IVS. Total placental volumetric blood flow was found to be significantly lower (p < 0.05) at G135 and G155 in THC-exposed versus control animals in post hoc comparison (Table 3). Placental oxygen availability was assessed using BOLD-MRI through analysis of quantitative placental *T*_2_* values. In control placentas at four timepoints across pregnancy, MR image voxels proximal to spiral artery sources of oxygenated maternal blood are characterized by relatively long *T*_2_* (Fig. 3), as previously described^35^. Concentration of deoxyhemoglobin is higher, reflected by decreased *T*_2_*, further from the spiral arteries, secondary to fetal oxygen uptake. There was a statistically significant smaller fraction of large *T*_2_* values in THC-exposed animals compared to controls across pregnancy (p = 0.04), demonstrating decreased placental perfusion and fetal oxygen availability in the former (Fig. 3).

**Table 3:** Dynamic contrast-enhanced MRI measurements.

Variable	Group	60 days	85 days	110 days	135 days	155 days
Total Placental blood flow (ml/min)^a^	Control	–	144.26 ± 51.6	169.91 ± 46.17	245.27 ± 52.06*	295.14 ± 36.79*
	THC	–	117.166 ± 57.5	160.58 ± 76.57	169.08 ± 54.35*	240.27 ± 40.53*
Normalized Placental blood flow (ml blood/ml placenta/min)^a^	Control	–	1.20 ± 0.18	1.20 ± 0.36	1.22 ± 0.03	1.26 ± 0.42
	THC	–	1.19 ± 0.35	1.11 ± 0.29	0.94 ± 0.05	1.07 ± 0.25

*p < 0.05.

^a^Obtained by DCE-MRI.

### Placental histology

**Figure 4:**
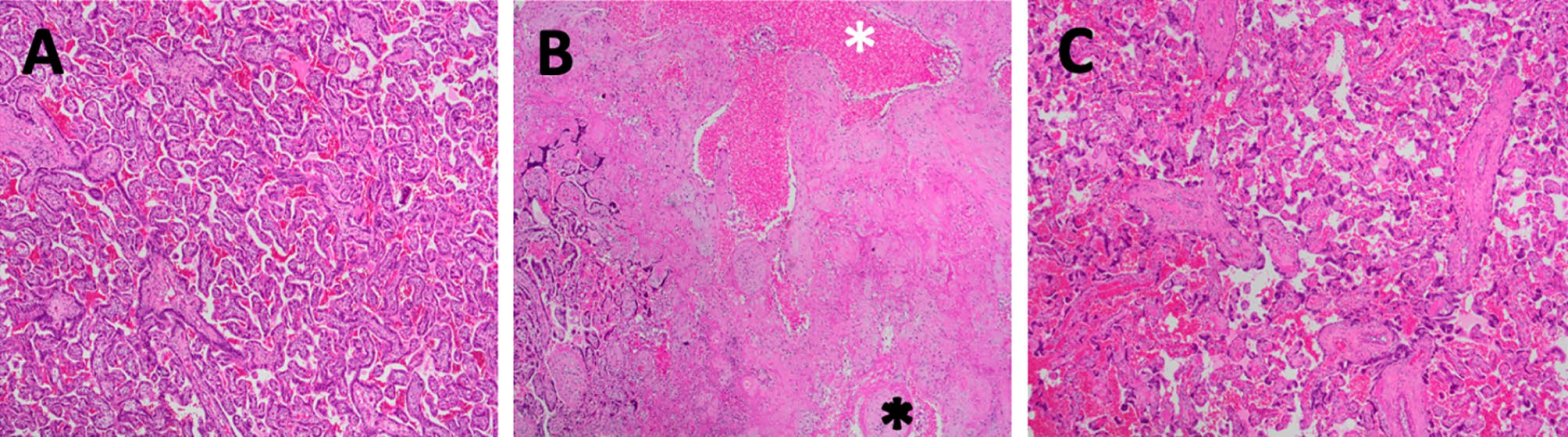
Placental microinfarctions associated with prenatal THC exposure. (**A**) Representative control H&E-stained placental section at gestational day 155 with normal villous and stroma, (**B**) acute (white asterix) and remote (black asterix) villous infarctions from a THC-exposed placenta, (**C**) markedly increased syncytial knotting from a THC-exposed placenta. Magnification is ×5.

Placental pathology demonstrated increased frequency of microscopic (< 1.0 cm) infarctions and syncytial knots in placentas exposed to THC (4/5, p < 0.05) compared with none in controls (0/5) (Fig. 4). There was no histologic evidence of infection, increase in placental villi maturation or findings of chorangiosis. Placental weights were not different between treatment groups (Table 2).

### RNA sequencing

**Figure 5:**
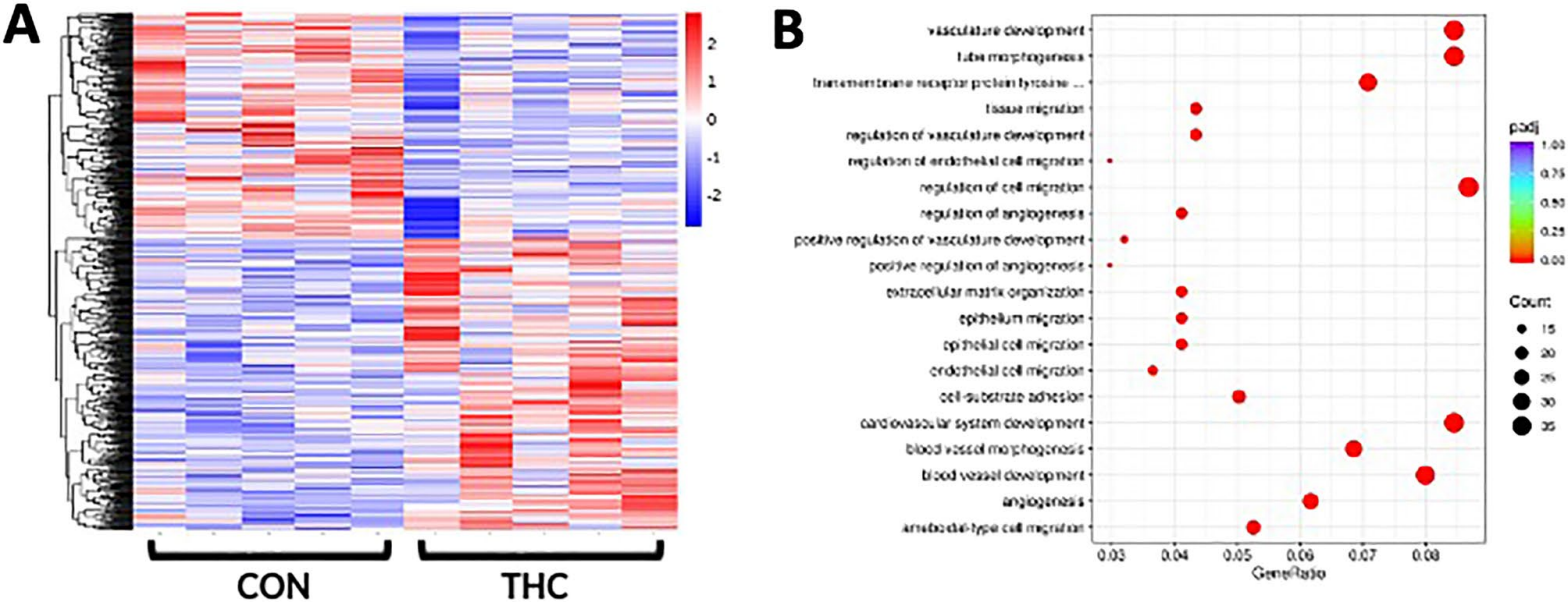
(**A**) Bulk placental RNA-sequencing suggests prenatal THC exposure is associated with a clear gene expression signature. RNA-sequencing of rhesus macaque placental tissue at gestational day 155. Heatmap of hierarchical clustering analysis of differentially expressed genes of control (left, n = 5) and THC-exposed (right, n = 5) placental samples. (**B) **RNA seq pathway analysis. Top significant terms in the Gene Ontology (GO) enrichment analysis of control versus THC-exposed placentas suggest prenatal THC exposure is associated with dysregulated placental vascular development and intracellular signaling, involved in angiogenesis.

Applying a false discovery rate analysis (p < 0.05), in THC-exposed compared to controls, we identified 426 upregulated genes and 327 downregulated genes (Supplemental Table 1). Hierarchical clustering analysis was performed on differentially expressed genes to identify a distinct gene expression signature in THC-exposed placentas when compared to control placentas (Fig. 5A). In THC-exposed placentas, we further identified 101 genes with p-value < 0.05 and fold-change > 1.5. Comparing THC-exposed placentas to control placentas, pathways with p < 0.001 are shown in Fig. 5B. Gene Ontology (GO) pathway analysis showed significant enrichment in DEGs involved in cytokine binding, regulation of cell migration, cell-substrate adhesion, angiogenesis, and vascular development (Fig. 5B). Similarly, KEGG pathway analysis showed enrichment in DEGs regulating cell migration.

## Discussion

Chronic prenatal THC exposure significantly diminished amniotic fluid volume, placental perfusion, and fetal oxygen availability throughout pregnancy, associated with altered placental pathology and transcriptome in the NHP. Histologic changes were notable for infarctions and consistent with gene expression enrichment in vascular developmental pathways. These data are all indicative of placental dysfunction resulting from chronic maternal THC use.

Previous animal studies have noted increased^22,56,57^ or decreased^58^ placental weight with prenatal cannabis exposure. Interestingly, in our study we did not find any differences in placental weight between treatment groups. This discrepancy may be due to the different route, dosage and timing of cannabis delivery studied. In prior animal studies, THC was often administered by intravenous, intraperitoneal, or oral gavage^59,60^, whereas our study used THC edibles to recapitulate typical human use^28^ and achieved plasma THC concentration ranges as previously reported in humans using similar weight-based dosing^31^.

Prior systematic reviews of human studies^18,61^ have reported a reduction in birth weight with prenatal cannabis exposure, but did not account for confounding factors such as tobacco and alcohol use. Our study did not find a difference in birth weight between THC exposed and control infants. A systematic review of human studies by Conner et al. adjusted for tobacco use and other confounding factors and also reported no increased risk of low birth weight with cannabis use in pregnancy^19^.

The clinical implications of our study are that our findings suggest that prenatal cannabis use is associated with placental insufficiency. The combination of significantly decreased placental perfusion and fetal oxygen availability, reduced amniotic fluid volume, and increased placental microinfarctions support a degree of placental dysfunction. The presence of oligohydramnios is a predictor of adverse perinatal outcomes, isolated oligohydramnios in the absence of fetal growth restriction has been associated with increased fetal and neonatal morbidity and mortality^62,63^. Thus, although fetal weight was maintained, other aspects of fetal development may have been disrupted.

This study was notable for a significant decrease in fetal testicular weight associated with maternal cannabis consumption. Although this finding was in a small sample, it is consistent with our published findings of a significant impact of chronic cannabis use on adult male testicular atrophy^25^. This is likely in part due to the presence of CB1 and CB2 receptors in both fetal and adult testes, suggesting that cannabis exposure can adversely affect male reproductive health, even in utero. Similar to a rat study of prenatal THC exposure^64^, we also observed a significant decrease in heart-to-body ratio at birth which may suggest longer term impairments in cardiac function in the offspring.

Our RNA sequencing findings were notable for involvement of angiogenesis and vascular pathways. Taken together with the histologic changes in THC-exposed placentas, these data suggest that prenatal THC exposure not only changes the structure of the placenta but also significantly alters gene expression in pathways regulating cell migration and vascular development. A recently published human cohort of maternal cannabis use reduced expression of placental genes involved in immune system function including type 1 interferon, neutrophil, and cytokine-signaling pathways^23^. Specifically, this study demonstrated decreased expression of immune genes, including hyperactivity-linked genes IL1B and CXCL8, and S100A8^23^. Similarly, our study was notable for increased expression of IL1RN, an IL1 receptor antagonist, and decreased expression of genes in the S100 protein family.

Our study’s strengths are that it utilizes a translational rhesus macaque model^29–31^ that imitates typical human cannabis consumption and provides the advantage of precisely measured THC-only exposure, avoiding the toxins of cannabis smoke, while retaining a contemporary, popular prenatal route of THC administration^26,28^. Other strategies previously used in animal studies^59,60^ (e.g., oral gavage, intravenous or intraperitoneal injections) may also introduce confounders associated with maternal stress^65^. The use of a NHP model also minimizes confounding from variables such as gestational age, quantity and timing of THC exposure throughout pregnancy. This model overcomes limitations in human studies including the ability to directly assess the effects of maternal THC consumption, longitudinal in vivo assessment with in utero MRI, and tissue studies. As THC is lipophilic, animals were selected to have a similar pre-pregnancy maternal weight in both treatment groups. All placentas were collected at time of cesarean section delivery prior to onset of spontaneous labor from pregnancies with similar environmental exposures, including diet. This controlled placental collection minimizes potential confounders, such as inflammation, that affect histologic evaluation. The longitudinal in-vivo imaging study design within the same pregnancy permits assessment of changes in placental perfusion and oxygenation across gestation. However, this study was limited by animal cohort size, which did not provide the power to examine fetal sex as a biological variable. With RNA sequencing analysis, our sample size did allow for the detection of DEGs with small effect sizes between treatment groups.

Although we observed placental perturbations from THC exposure, the underlying mechanisms for these observations are not well understood. Maternal environmental exposures ranging from pregnancy nutrition, to opioids and smoking have all been associated with an altered placental transcriptome, including changes linked to neurodevelopmental disorders^66–70^. THC exerts its effects via receptors of the endocannabinoid system, which plays a critical role in mediating placental and fetal development^11–15,71^. Endocannabinoid receptors are present in the placenta and major fetal organs, including the brain, starting early in pregnancy^72^. Although we did not observe a THC effect on fetal brain weights, ongoing work from our group is focused on the impact of prenatal THC on offspring sociobehavior and neurodevelopment. Our RNA sequencing findings reveal mechanistic targets for future investigation to develop interventions or treatments in pregnancies affected by maternal cannabis use. Although contemporary cannabis products used in pregnancy can also contain high doses of cannabidiol (CBD), we chose to study the direct effects of THC only as an initial step given it is the main psychoactive component of cannabis. Future studies will focus on the impact of CBD only and commonly used ratios of THC to CBD.

## Summary

The long-term consequences of the adverse placental outcomes we report are unknown, but may impact offspring development. The findings of this study contribute to the limited existing safety data on prenatal THC exposure. At this time, it is important for healthcare providers to counsel pregnant individuals to abstain from THC use until further research is conducted.

## Supplementary Information

Supplementary Table 1.

## Data Availability

The raw and processed RNA-seq datasets used and/or analyzed during the current study are publicly accessible through NCBI Gene Expression Omnibus (GEO) via accession series GSE216112.
